# Higher VTE in a Roma population attributed to raised genetic risk and gene-environmental interaction

**DOI:** 10.1093/eurpub/ckac131.135

**Published:** 2022-10-25

**Authors:** S Natae, J Sándor, R Ádány, S Fiatal

**Affiliations:** 1 Department of Public Health and Epidemiology, University of Debrecen, Debrecen, Hungary; 2 Doctoral School of Health Sciences, University of Debrecen, Debrecen, Hungary; 3 MTA-DE Public Health Research Group, University of Debrecen, Debrecen, Hungary

## Abstract

**Background:**

Interactions between genetic and environmental risk factors (GxE) contribute to an increased risk of venous thromboembolism (VTE). Understanding how these factors interact provides insight for the early identification of at-risk groups within a population and creates an opportunity to apply appropriate preventive and curative measures.

**Objective:**

To estimate and compare GxE for VTE risk in the general Hungarian and Roma populations.

**Methods:**

The study was based on data extracted from a database consisting the results obtained previously in a three pillars complex health survey. DNA was genotyped for rs121909567 (SERPINC1), rs1799963 (F2), rs2036914 (F11), rs2066865 (FGG), rs6025 (F5), and rs8176719 (ABO) polymorphisms. Multivariable linear regression analysis was applied to test the impact of GxE on VTE risk.

**Results:**

Interestingly, the rs121909567 (SERPINC1) SNP was not present in the general population, however the risk allele frequency was 1.4% among Roma, which might suggest a founder effect in this minority. The risk of VTE was higher among depressive Roma subjects who carried the risk variant of rs2036914 (β = 0.819, p = 0.02); however, not for the general subjects. The joint presence of high level of LDL-C and rs2066865 increased the VTE risk only among Roma (β = 0.389, p = 0.002). A multiplicative interaction between rs8176719 and cancer was identified and higher for the Roma population (β = 0.370, p < 0.001). The VTE risk increased in the Roma population (β = 0.280, p = 0.001) but was higher in the general population (β = 0.423, p = 0.001) as a result of the multiplicative interaction between CAD and rs2036914 (F11).

**Conclusions:**

rs121909567 (SERPINC1) was confirmed as a founder mutation in the Roma population. As a result of higher genetic load and GxE interactions, the minority Roma population is at higher risk of VTE than the general Hungarian population.

**Key messages:**

• Our study revealed some evidence on the burden of the joint presence of genetic and environmental risk factors on VTE.

• A marginalized and segregated Roma community could need due attention for the prevention and control of CVDs.

